# Discovery of RUVBL1 as a Target of the Marine Alkaloid Caulerpin via MS-Based Functional Proteomics

**DOI:** 10.3390/md24010037

**Published:** 2026-01-10

**Authors:** Alessandra Capuano, Gilda D’Urso, Lucia Capasso, Emilio Brancaccio, Erica Gazzillo, Marianna Carbone, Ernesto Mollo, Gianluigi Lauro, Maria Giovanna Chini, Giuseppe Bifulco, Angela Nebbioso, Agostino Casapullo

**Affiliations:** 1 Department of Pharmacy, University of Salerno, Via Giovanni Paolo II 132, 80084 Fisciano, Italy; acapuano@unisa.it (A.C.); gidurso@unisa.it (G.D.); embrancaccio@unisa.it (E.B.); egazzillo@unisa.it (E.G.); glauro@unisa.it (G.L.); bifulco@unisa.it (G.B.); 2 Department of Precision Medicine, University of Campania “Luigi Vanvitelli”, Via De Crecchio 7, 80138 Naples, Italy; lucia.capasso2@unicampania.it (L.C.); angela.nebbioso@unicampania.it (A.N.); 3 Institute of Biomolecular Chemistry, National Research Council of Italy, Via Campi Flegrei 34, 80078 Pozzuoli, Italy; marianna.carbone@cnr.it (M.C.); emollo@icb.cnr.it (E.M.); 4 Department of Biosciences and Territory, University of Molise, Contrada Fonte Lappone, 86090 Isernia, Italy; mariagiovanna.chini@unimol.it

**Keywords:** alkaloid, caulerpin, limited proteolysis, mass spectrometry, RUVB-like1

## Abstract

Marine flora is a significant source of bioactive metabolites. These compounds have been demonstrated to have outstanding bioactivity and biocompatibility, enabling their use in various therapeutic applications. Therefore, examining the biological potential of marine natural compounds remains important, with particular emphasis on their interaction profiles to identify the macromolecular partners they can modulate. This study focused on the interactome profiling of the marine alkaloid caulerpin (CAU), isolated from the alga *Caulerpa cylindracea*. Along with the discovery of its antitumor properties, this metabolite has garnered attention for its potential therapeutic applications, including modulation of MAO-B and PPARs involved in inflammatory responses, as well as the discovery of its antitumor properties. Two complementary MS-based proteomic approaches were used to identify CAU target proteins in cancer cells: DARTS, which enabled proteome-wide screening to identify proteins interacting with the compound, and t-LIP-MRM-MS, which pinpointed the target protein regions involved in ligand binding. RUVB-like 1 (RUVBL1), a protein that regulates the essential mechanism of carcinogenesis, including chromatin remodeling, DNA repair, and transcriptional control, was discovered as an intriguing CAU target. These results were corroborated via in silico and biological investigations that elucidated CAU role in the regulation of RUVBL1 activity, highlighting its promising therapeutic relevance.

## 1. Introduction

In the field of drug discovery, metabolites are no longer just metabolic byproducts; they represent nature’s own hit compounds [1] that can interact with new biological targets, guiding innovative and effective drug development.

The ocean’s potential is evident in the remarkable variety of organisms living in this unique environment. Sponges, corals, and other marine organisms produce several secondary metabolites as part of their natural defense mechanisms. These metabolites are characterized by wide structural diversity and exhibit interesting biological properties relevant to human health, such as antibacterial, antiviral, anticancer, and immunomodulatory activities [2].

The study of these metabolites can lead to the discovery of promising hit compounds, harnessing both their chemical diversity and biological potential to develop innovative therapies [3].

Alkaloids represent one of the most widespread classes of natural products (NPs); they comprise around 20% of all plant secondary metabolites, exhibit interesting chemical and biological features, and are found in both terrestrial and marine organisms [4]. These metabolites, mostly derived from amino acids, are chemically characterized by heterocyclic structures containing nitrogen atoms, which confer them specific acid–base properties [5,6].

Furthermore, alkaloids have traditionally been a central focus of scientific inquiry owing to their broad spectrum of biological and pharmacological properties, encompassing anti-inflammatory, antimicrobial, cardioprotective, and analgesic effects [7]. A significant milestone was achieved in the 1950s when Canadian researchers Charles Beer and Robert Noble isolated the first *Vinca* alkaloids, thereby initiating comprehensive research into their anticancer capabilities [8].

**Figure 1:**
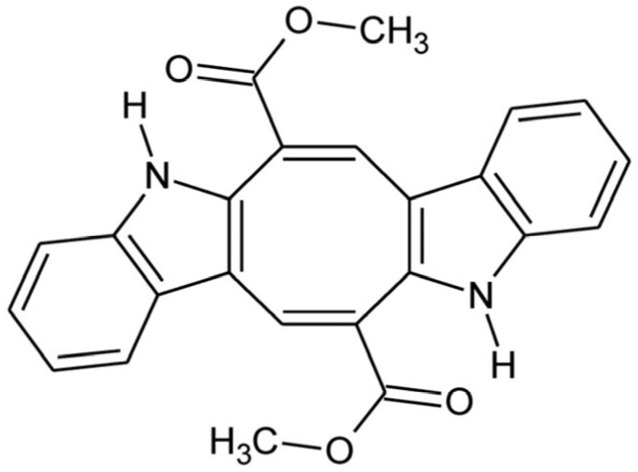
Caulerpin: chemical structure.

Over the past three decades, a growing number of marine alkaloids derived from sponges, tunicates, and algae have shown anticancer potential, becoming essential components of modern anticancer research [4]. Caulerpin (CAU), a symmetric bis-indole alkaloid containing an eight-carbon ring fused with the two indole units and substituted with two carbonyl groups (Figure 1) [5], is isolated from some marine green algae of the genus *Caulerpa* [6,7], comprising *C. cylindracea,* an Indo-Pacific species that has become highly invasive to the Mediterranean Sea [9].

CAU has demonstrated significant biological activities, including antinociceptive, anti-inflammatory [7], and antimicrobial properties against foodborne pathogens [10], antituberculosis effects [11,12], and promising hypolipidemic action [13]. This compound is also known to act as an agonist of peroxisome proliferator-activated receptors (PPARs) [14] and also exhibits cytotoxic activity against multiple human cancer cell lines, with IC_50_ values ranging from 0.72 to 4.67 µM [15]. Although CAU’s anticancer activity was previously evaluated by measuring its cytotoxicity on several human cancer cell lines [15,16,17], current knowledge remains fragmented, and its mechanism of action is unclear.

Given the promising biological properties of CAU, this study focused on analyzing its interaction profile within a human cell proteome to pinpoint its potential protein targets and elucidate the pathways involved in the reported cytotoxic activity. To this end, we decided to adopt an experimental approach that has previously been proven to be useful and successful for profiling the interaction patterns of natural small metabolites [18]. The workflow integrates two label-free functional proteomics techniques based on limited proteolysis: Drug Affinity Responsive Target Stability (DARTS) and targeted Limited Proteolysis coupled with Mass Spectrometry (t-LiP-MS). Both approaches are based on protein structure stabilization after ligand binding. The binding moves the protein conformation towards a more compact state that is less sensitive to limited proteolysis induced by a nonspecific protease compared to the free protein. DARTS enables the identification of the molecule’s target proteins in a cell lysate [19,20], whereas t-LiP-MS enables the recognition of the target protein regions involved in the binding, providing details about the ligand–protein complex at a molecular level [21,22]. The DARTS analysis performed in this study identified RUVB-like 1 (RUVBL1), an eukaryotic protein that belongs to the AAA+ ATPase family [23,24], as the most promising candidate, due to its key role in cancer.

Its function is closely linked to its paralogue RUVBL2, as their interaction leads to a double hexameric structure, endowed with ATPase activity [25]. RUVBL1 is involved in a broad spectrum of cellular pathways [26], some of which play crucial roles in cancer processes, such as chromatin remodelling [20,21] and the regulation of transcriptional oncogenic factors [27]. Moreover, RUVBL1 is overexpressed in various types of cancer, including non-small cell lung cancer [28], head and neck cancers, and pancreatic ductal adenocarcinoma [29], and its overexpression has also been associated with increased invasiveness and therapy resistance in certain types of cancer.

Given its role in tumorigenesis and the potential relevance of RUVBL1 as an anticancer target, we sought to investigate this protein further using the t-LiP technique, which is a complementary approach that enabled the identification of five different protein regions involved in CAU binding. These results were corroborated via molecular docking experiments and molecular dynamics simulations, which highlighted the capacity of CAU to interact with the RUVBL1 ATP binding site. After that, biological assays were used to evaluate the cell viability, proliferation, cell cycle distribution, and mitochondrial activity upon treatment with CAU. The cytotoxic effects and reduction in the total cellular ATP production were highlighted, confirming the involvement of CAU in the RUVBL1’s mechanism of action and suggesting CAU’s potential applications in future cancer treatments.

## 2. Results and Discussion

### 2.1. Discovering CAU Protein Counterparts Through Drug Affinity Responsive Target Stability (DARTS)

DARTS studies were performed in triplicate, utilizing a lysate of HeLa tumor cells to examine CAU’s interaction profile.

The protein extract was divided into aliquots, and different concentrations of CAU (1 µM, 10 µM, and 100 µM) were added to separate aliquots, with one aliquot that received only the vehicle serving as a reference.

After this incubation, the proteins underwent limited proteolysis with various enzyme-to-protein ratios, followed by separation through SDS-PAGE electrophoresis. After Coomassie staining, the gel was divided into distinct bands based on molecular weight, and these gel pieces were removed for thorough digestion with trypsin to prepare samples for the following bottom-up mass spectrometric analysis [18], using a nano-flow UPLC system Ultimate 3000 connected to the nano-ESI source of a Thermo Q-Exactive mass spectrometer (Thermo Fisher Scientific, Bremen, Germany).

To generate a peak list providing comprehensive mass spectrometry information about peptide mixtures, the acquired data were initially processed with Xcalibur software version 2.2 and then analyzed using Proteome Discoverer (PD) version 2.4 in conjunction with the SwissProt Human database. This approach enabled protein identification by matching experimental results with entries in the SwissProt database. Moreover, a relative quantification was performed by comparing protein abundances in the CAU-treated samples versus those in the reference sample. Three proteins showed a significant increase in abundance in the CAU-treated samples (see Supplementary Materials Table S1). Among them, we chose to focus initially on RUVBL1 (Table 1) because of its role as a regulatory node within several pathways crucial to cancer biology. In fact, RUVBL1 is essential for assembling and operating key chromatin-remodeling complexes. Additionally, although Kinetochore protein Spc24 and Serine/threonine-protein phosphatase 2A are also involved in tumor-related processes [30,31], RUVBL1 is the most extensively characterized in this area, thereby underscoring the heightened need for new scaffolds capable of modulating this specific target [32], which prompted our decision to focus on this target molecule, which we analyze extensively in this study.

### 2.2. Western Blot Analysis of RUVBL1 in the Presence of CAU

**Figure 2:**
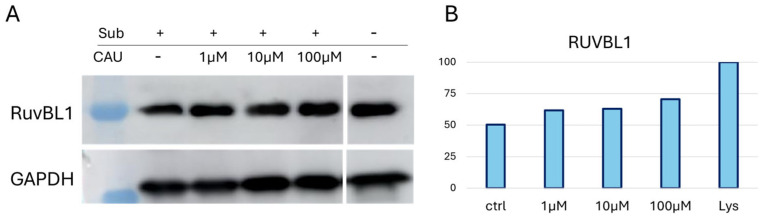
(**A**) One WB analysis of DARTS samples showed the increasing abundance of RUVBL1 upon CAU interaction. (**B**) Graphical representation of anti-RUVBL1 signals normalized on GAPDH. “Lys” is the positive control relative to the protein extract without treatment (no CAU and no limited proteolysis), set to 100%.

Samples from a representative DARTS replicate were subjected to Western blot analysis using an anti-RUVBL1 antibody to confirm further the mass spectrometric DARTS trend (Figure 2). GAPDH (Glyceraldehyde 3-Phosphate Dehydrogenase) was employed as an internal loading control, since it is a ubiquitously expressed and widely recognized housekeeping protein that ensures normalization of protein loading across the lanes. Notably, GAPDH is relatively stable against protease digestion, making it particularly suitable for this type of analysis [33]. Following the blotting procedure, ImageJ software version 1.52 a (NIH, Bethesda, MD, USA) was used for graphic representation of RUVBL1 bands’ intensities in the tested samples (Figure 2B) across different experimental conditions (Figure 2). A CAU-dependent increase in RUVBL1 signal supported the trend observed in MS-DARTS experiments.

### 2.3. t-LiP-MS-Guided into the Conformational Features Underlying the Interaction Between CAU and RUVBL1

Following the identification of RUVBL1 as the selected cellular partner of CAU, our objective was to elucidate the molecular basis of their interaction. To this end, we employed the t-LiP-MS method. Given that ligand binding enhances protein stability (thereby diminishing susceptibility to proteolytic enzymes and revealing potential binding sites), this technique involves analyzing the tryptic peptides derived from the target protein after its treatment with a nonspecific protease under limited proteolysis conditions. As trypsin exhibits high specificity, the prediction of the resultant peptide fragments is straightforward. Subsequently, Multiple Reaction Monitoring-Mass Spectrometry (MRM-MS) is utilized to monitor these fragments. MRM-MS characterizes peptides based on their *m*/*z* ratios and distinctive fragmentation patterns. When limited proteolysis occurs before tryptic digestion, certain peptides may be cut by a nonspecific enzyme and therefore remain undetectable. Conversely, if a ligand protects these regions, the cleavage sites are maintained, and the protected tryptic peptides can be detected again. By comparing peptide abundances between the ligand-treated and control samples, regions with increased protection can be identified and interpreted as potential binding sites.

The design of the Multiple Reaction Monitoring (MRM) experiment began with the selection of RUVBL1 tryptic peptides using a computer-aided approach. The *m*/*z* values for 18 RUVBL1 tryptic peptides were obtained from the PeptideAtlas Human database, and for each of them, the three most reliable fragment ions (transitions) were selected from the Complete Human SRMAtlas build. The first test experiment was performed on a tryptic digest of HeLa lysate, and only the best transition for each peptide was chosen, resulting in a final panel of 12 transitions (Table S2).

In the t-LiP-MRM experiments, HeLa lysates were incubated under native conditions with either 100 μM CAU or DMSO (control) for one hour at room temperature. The samples were then subjected to limited proteolysis followed by extensive digestion with trypsin. The previously optimized methods were used to analyze the resulting peptide mixtures. This comparison was carried out in triplicate and led to the identification of five RUVBL1 peptides affected by ligand binding, with a fold change greater than 1.5 and a *p*-value < 0.05 (Table 2).

The identified peptides map to critical functional motifs of RUVBL1. Specifically, they involve the Walker A motif (P-loop, aminoacidic residues 70–79), essential for ATP phosphate coordination [34], and a region located in proximity to the Sensor 2 site (~397–406), which transduces ATP hydrolysis into conformational changes [26].

Considering the significance of these regions, the results suggested that CAU binding could substantially influence the enzyme’s catalytic activity by affecting its conformational dynamics. These pivotal findings are promising and have prompted additional investigations to scrutinize this possibility in greater detail.

### 2.4. t-LiP-MS Corroboration: Molecular Docking and Molecular Dynamics Simulations

**Figure 3:**
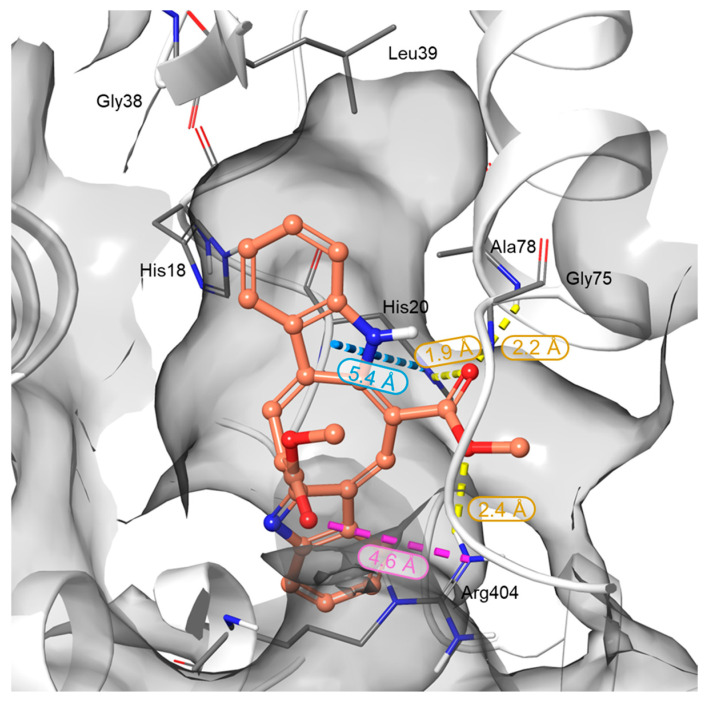
Three-dimensional representation of the CAU binding mode within the RUVBL1 binding site. RUVBL1 is shown as a gray surface, while CAU atoms are colored by elements: carbon in orange, oxygen in red, and nitrogen in blue. H-bonds, salt bridges, and π-π interactions are represented in yellow, violet, and cyan dotted lines, respectively. Bond distances are reported next to each interaction and fall within the default criteria defined in Maestro (Schrödinger Suite).

**Figure 4:**
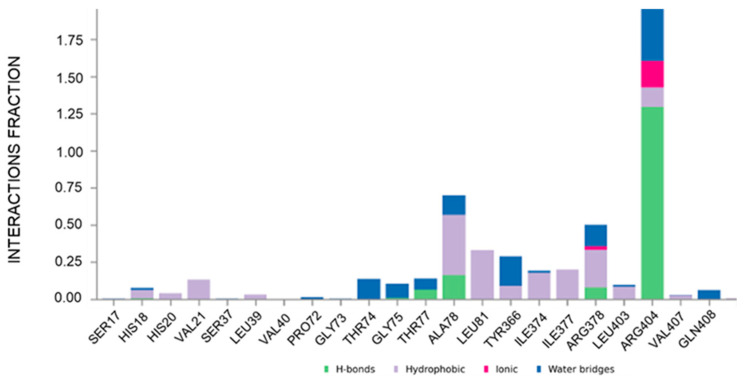
Plot of the interactions maintained throughout the 500 ns simulation (CAU-RUVBL1). Values over 1.0 (100%) are possible as some protein residue may form multiple contacts of the same subtype with the ligand.

In silico studies were performed to elucidate the binding behavior of CAU toward RUVBL1 and to corroborate the t-LiP results. In detail, RUVBL1 is an evolutionarily conserved AAA+ ATPase that participates in large multiprotein assemblies, including the INO80 and TIP60 chromatin remodelling complexes [26]. It frequently forms a heterohexameric complex with its close homolog, RUVBL2. Several crystal structures of this hexamer are available in the Protein Data Bank (PDB), which served as the starting point for the structural analysis of CAU–RUVBL1 interactions [34,35]. Guided by the protected peptides identified in chemoproteomic experiments, the ATP-binding site of RUVBL1 was recognized as the primary region engaged by CAU. This pocket is a highly conserved nucleotide-binding cavity typical of AAA+ ATPases, containing canonical motifs essential for ATP recognition and hydrolysis: the Walker A motif (Gly70-Thr77), which is a glycine-rich segment (GXXGXG) with a conserved lysine residue that coordinates the phosphate groups of ATP; the Walker B motif (Asp302-His305), which contributes to Mg^2+^ coordination and catalyzes the ATP hydrolysis; the Sensor 1 region (Phe329-Asp332), which detects the γ-phosphate of the nucleotide, and Sensor 2 (Thr401-Ser406), which mediates communication between the ATPase active site and protein conformational changes (Figure 3) [36,37]. Taken together, the structural and chemoproteomic data strongly suggest that CAU engages the ATP-binding pocket of RUVBL1. In agreement with this hypothesis, molecular docking studies revealed specific interactions within this region, showing that CAU forms a salt bridge with Arg404 (Sensor 2 region), a hydrogen bond with Ala78, and a π–π stacking interaction with His18 in the N-terminal region, which is also crucial for ATP binding. Additional hydrophobic contacts were observed, consistent with the peptides identified by t-LiP analysis (Figure 4).

To gain molecular-level insight into the structural determinants governing CAU-RUVBL1 interaction, molecular dynamics (MD) simulations were performed. The ligand protein complex was first subjected to energy minimization and equilibration under NVT and NPT ensembles, followed by a 500 ns production run (see Section 3). Analysis of the MD trajectories indicated that CAU maintained stable interactions with the following residues: His18, His20, Val21, Leu39, Thr74, Gly75, Thr77, Ala78, Leu81, Tyr366, Ile374, Ile377, Arg378, Leu403, Arg404, Val407, and Gln408, throughout the simulation (interaction occupancies between ~10% and ~190%, where values over 100% indicate multiple contacts between a given protein residue and the ligand, Figure 4). These residues belonging to conserved motifs are crucial for ATP binding and hydrolysis, thus corroborating that CAU targets functionally important regions of RUVBL1. Their involvement, consistent with both computational and chemoproteomic findings, supports the proposed CAU-RUVBL1 binding mode.

### 2.5. Biological Effect of CAU in HeLa Cells

#### 2.5.1. Cytostatic Activity of CAU in HeLa Cells

**Figure 5:**
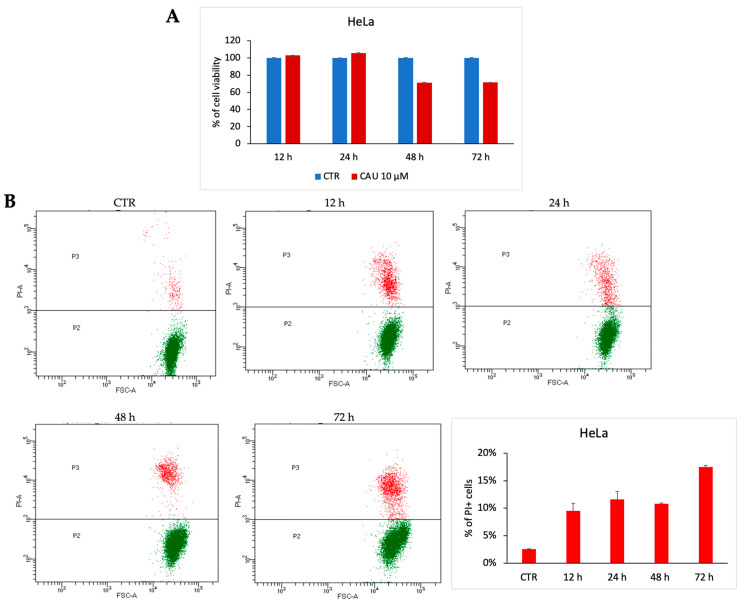
CAU induces cytostatic effect in HeLa cells. HeLa cells were treated with CAU (10 µM) or left untreated (control, CTR) and analyzed at multiple time points. (**A**) Cell viability was assessed by MTT assay at 24, 48, and 72 h. Results are expressed as the percentage of viable cells relative to control (mean ± SD, *n* = 3). (**B**) Representative dotplot of HeLa cells treated with CAU 10 µM and analyzed at multiple time points. The percentage distribution of PI+ cells was plotted in the graph below (mean ± SD, *n* = 2).

To evaluate the potential antiproliferative effect of 10 µM CAU in HeLa cells, cell viability was assessed through the MTT assay at different time points (24-, 48-, and 72-h post-treatment). The data revealed that CAU induces only a slight decrease in cell viability, with no evidence of significant cytotoxicity (Figure 5A), thereby supporting a predominantly cytostatic behavior. These findings were further confirmed by a dose-dependent analysis of CCK-8 (Supplementary Figure S1).

For a deeper investigation of this biological effect of CAU, PI-positive cells were quantified post-treatment. Prolonged exposure resulted in a slight increase in late cell death events, in line with a cytostatic rather than a robust cytotoxic response (Figure 5B). Cell cycle analysis at 72 h supported this conclusion (Supplementary Figure S2).

Taken together, these results suggest a cytostatic activity of CAU in HeLa cells, characterized by a modest reduction in cell viability accompanied by a delayed onset of cell death.

#### 2.5.2. CAU Reduces ATP Production in HeLa Cells

**Figure 6:**
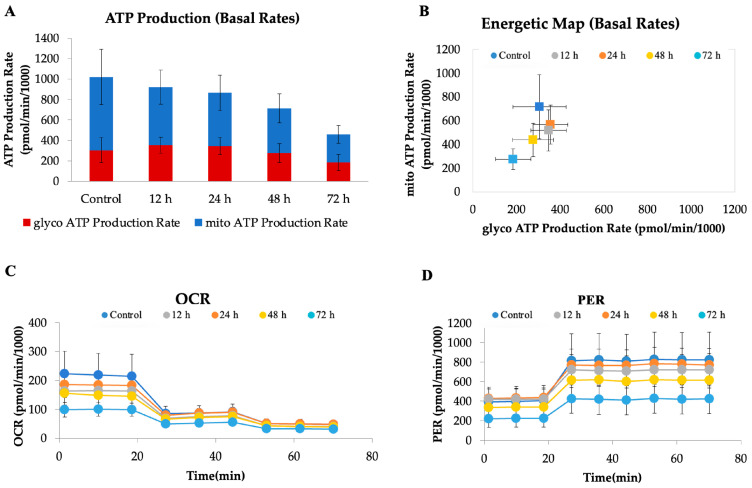
CAU treatment disrupts cellular bioenergetics in HeLa cells. HeLa cells were treated with CAU (10 µM) for 12, 24, 48, and 72 h before analysis. (**A**) ATP production rates from mitochondrial and glycolytic sources were measured using the Seahorse XF Real-Time ATP Rate Assay. (**B**) Energy map generated from OCR and PER values. (**C**) Oxygen consumption rate (OCR) profile measured using the ATP Rate Assay Kit, with sequential injections of oligomycin (1.5 µM), and rotenone/antimycin A (0.5 µM each). (**D**) Proton efflux rate (PER) values were derived according to the manufacturer’s ATP Rate Assay protocol. Data are shown as mean ± SD from four replicates.

To investigate the metabolic impact of CAU (10 µM) on HeLa cells, a real-time bioenergetic profile was assessed using the Seahorse XF Analyzer. This platform enables the simultaneous measurement of mitochondrial respiration and glycolytic activity through the oxygen consumption rate (OCR) and proton efflux rate (PER), respectively. As shown in the ATP production rate assay (Figure 6A), CAU-treated cells exhibited a marked reduction in total ATP production, affecting both mitochondrial and glycolytic contributions. This global suppression of energy metabolism indicates a profound metabolic collapse. The energetic map (Figure 6B), which integrates OCR and PER values, further supports this finding: while control cells display a highly active metabolic phenotype, CAU-treated cells cluster in a region consistent with quiescence or metabolic stress. The OCR profile (Figure 6C) revealed a dramatic inhibition of mitochondrial respiration following CAU exposure, as shown by the decrease in basal and maximal respiration. Concurrently, the PER profile (Figure 6D) demonstrated a sharp reduction in extracellular proton efflux, indicating suppressed glycolytic flux. Notably, treated cells did not increase PER upon glycolytic stimulation, suggesting severely impaired metabolic flexibility. Together, these data indicate that CAU exerts a dual inhibitory effect on oxidative phosphorylation and glycolysis, leading to a drastic drop in ATP availability and compromising cellular energy homeostasis.

## 3. Materials and Methods

### 3.1. Drug Affinity Responsive Target Stability

Pure CAU was isolated from a sample of *C. cylindracea* collected in the Gulf of Pozzuoli (Italy), following previously reported procedures [38]. The compound was identified by comparing ^1^H- and ^13^C-NMR spectroscopic data with literature values [12].

HeLa cells were cultured at 37 °C in a 5% CO_2_ atmosphere using Dulbecco’s Modified Eagle Medium supplemented with 10% (*v*/*v*) fetal bovine serum, 100 U/mL penicillin, and 100 mg/mL streptomycin (Sigma-Aldrich, St. Louis, MO, USA). The cells were subsequently collected via centrifugation at 1000× *g* for 5 min. They were suspended in PBS containing 0.1% Igepal and a protease inhibitor cocktail (GeneSpin, Milan, Italy). Mechanical lysis was performed using a Dounce homogenizer at 4 °C. The homogenate was centrifuged at 10,000× *g* for 5 min at 4 °C to remove debris. The supernatant was collected, and protein concentration was measured using the Bradford assay (Bio-Rad, Hercules, CA, USA). Four aliquots were prepared, incubated with CAU (1 µM, 10 µM, and 100 µM), along with a DMSO control. After 1 h at room temperature with agitation (Mini-Rotator, Biosan, Riga, Latvia), samples were divided for proteolysis with subtilisin at ratios of 1:1500 and 1:500 *w*/*w* for 30 min at 25 °C with shaking at 500 rpm. One aliquot remained undigested as a control. PMSF at 1 mM (Sigma-Aldrich, St. Louis, MO, USA) was used to stop digestion. The experiment was performed in triplicate. For electrophoretic separation, 20 µg of protein from each sample was mixed with an SDS-PAGE loading buffer (60 mM Tris/HCl, pH 6.8, 2% SDS, 0.001% bromophenol blue, 10% glycerol, 2% 2-mercaptoethanol) and heated at 95 °C for 5 min. The mixture was then loaded onto a 4–12% Bis-Tris Criterion XT Precast Gel (Bio-Rad Laboratories, Hercules, CA, USA). Electrophoresis was performed using a Bio-Rad apparatus. The gel was fixed for 15 min in a fixing solution (50% H_2_O, 40% MeOH, 10% AcOH). Afterwards, it was washed three times with water, stained with Coomassie (Bio-Rad Laboratories, Hercules, CA, USA) for 1 h at room temperature with continuous shaking (see Supplementary Figure S3), and then divided into different bands for in situ digestion with trypsin [25]. The solutions containing tryptic peptides were dried using a SpeedVac (Eppendorf, Hamburg, Germany) and then redissolved in 30 µL of 10% formic acid for nano-flow RP-UPLC MS/MS analysis. Subsequently, 1 µL of each sample was analyzed via Q-Exactive Classic MS (Thermo Fisher Scientific, Bremen, Germany) coupled with an UltiMate 3000 UPLC system (Thermo Fisher Scientific, Bremen, Germany), using an EASY-Spray Pepmap™ RSLC C18 column (Thermo Fisher Scientific, Bremen, Germany) with a gradient from 1 min at 3% B to 60 min at 95% B (A: 95% H_2_O, 5% CH_3_CN, 0.1% acetic acid; B: 95% CH_3_CN, 5% H_2_O, 0.1% AA). Capillary temperature was set at 250 °C, and gas flows were set to 0, with an AGC target of 3 × 10^6^, and a 50 ms ion transfer time. MS spectra were acquired over a *m/z* range of 375–1500 at a resolution of 70,000. Data-dependent MS/MS scans were performed with the following settings: 8 loops, 17,500 resolution, an AGC target of 1 × 10^5^, up to 80 ms IT, a collision energy of 26%, and an isolation width of 1.6. Raw data were analyzed with Proteome Discoverer 2.4 using SwissProt (UniProt Consortium) and Sequest HT (Thermo Fisher Scientific, Bremen, Germany), with in silico matching and Percolator validation, allowing up to two missed cleavages, fixed carbamidomethyl modification, and variable oxidation and N-terminal acetylation. Proteomic analysis of individual raw files provided protein abundance values for each sample, and the fold changes were calculated as a ratio between treated samples and the corresponding untreated control, thereby revealing differences in residual protein abundance following limited proteolysis. Proteins displaying a progressive increase in fold change in accordance with increasing amounts of the compound were selected as putative targets.

### 3.2. Western Blotting for DARTS Validation

The DARTS sample was subjected to electrophoresis in a 10% SDS-PAGE gel. Proteins were transferred onto a nitrocellulose membrane, which was incubated in 5% non-fat dried milk with T-TBS (31 mM Tris, pH 8, 170 mM NaCl, 3.35 mM KCl, 0.05% Tween 20) for 1 h at room temperature with shaking. The membrane was then incubated overnight at 4 °C with a primary mouse antibody against RUVBL1 (1:1000, Santa Cruz Biotechnology, Dallas, TX, USA), followed by a mouse peroxidase-conjugated secondary antibody (1:1000, Thermo Fisher Scientific, Bremen, Germany). Signals were developed using enhancer and peroxide solutions (GeneSpin, Milano, Italy) and detected with a LAS4000 (GE Healthcare, Chicago, IL, USA) system. The procedure was repeated with a primary anti-GAPDH antibody (1:2500, Santa Cruz Biotechnology) to control gel loading.

### 3.3. Targeted Limited Proteolysis

PeptideAtlas and SRMAtlas were utilized to select RUVBL1 (UniProt code: Q9Y265) tryptic peptides and their fragment ions. The top three transitions per peptide were chosen to develop MRM methods, which were then tested on a tryptic-digested HeLa lysate [13]. The peptide mixture was dissolved in 10% FA, and 30 µg of the peptide mixture was injected into an Aeris^TM^ 3.6 µm PEPTIDE XB C18 column (50 × 2.1 mm) (Phenomenex, Torrance, CA, USA), employing a gradient from 5% to 95% B over 20 min (A: 0.1% FA in H_2_O, B: 0.1% FA in CH_3_CN). The 6500 Q-Trap used ion spray for detection via MRM in positive mode. The QTrap settings were as follows: CUR = 30; IS = 5500; TEM = 250 °C; GS1 = 25; GS2 = 25; DP = 80; EP = 15; CXP = 12. Data were analyzed with Analyst 1.6.2.

The interaction study of the RUVBL1-CAU complex was conducted by incubating HeLa cell lysate with CAU at 100 µM or DMSO for 1 h at room temperature with shaking (Mini-Rotator, Biosan). Subtilisin (enzyme-to-protein ratio 1:1500) was applied for 30 min at 25 °C under continuous shaking (500 rpm, Thermomixer, Biosan). As a control, an undigested sample without the molecule was maintained. After quenching the enzyme with PMSF (final concentration of 1 mM), samples were denatured with 4 M urea and digested in solution with trypsin. MRM-MS analysis was performed in triplicate using the optimized RUVBL1 MRM methods. The tryptic peptide peak regions were measured using Analyst Software (AB Sciex, Foster City, CA, USA).

### 3.4. Computational Details

The Schrödinger Suite 2025-2 (New York, NY, USA) was employed to conduct the computational analyses described below.

#### 3.4.1. Molecular Docking Experiments

The crystallographic structure of RUVBL1 (PDB ID: 9EMA [39]) was employed as a starting model for docking studies. This structure contains six ATP molecules, namely three bound to the nucleotide-binding sites of RUVBL1 and three to those of RUVBL2, as well as three molecules of the allosteric inhibitor CB-6644. Based on insights from chemoproteomic analyses, one ATP molecule located in the RUVBL1 binding pocket was selected as the reference for grid box definition. The protein complex was prepared using the Protein Preparation Workflow (included in Schrödinger Suite), considering a pH ranging from 6.4 to 8.4, by adding missing hydrogen atoms, assigning proper bond orders, and subsequently removing all water molecules. The docking grid was centered on the active site region (coordinates in Å: X = 148.72, Y = 96.20, Z = −105.86) with inner box dimensions of 10 Å and outer box dimensions of 25.40 Å. The 2D structure of CAU was built using Maestro 2D sketcher (Schrödinger Suite) and processed with LigPrep (Schrödinger Release 2025-2: Ligprep, New York, NY, 2025) to generate possible tautomers, assign the protonation state at pH 7.4 ± 1.0, and optimized geometry using the OPLS 2005 force field. Docking calculations were carried out in Glide [40,41,42,43] (Schrödinger Release 2025-2: Glide version 10.7, New York, NY, 2025) using the Extra Precision (XP) mode within the Schrödinger Suite. For the ligand, 10 poses were generated to ensure thorough sampling of potential binding conformations. The most favorable docking poses were selected through visual inspection, considering both the docking score and key interactions with critical amino acid residues in the binding site.

#### 3.4.2. Molecular Dynamics Simulations

The selected docking pose of CAU in the RUVBL1 binding site (PDB ID: 9EMA [39]) was used as the starting point for molecular dynamics (MD) simulations, performed in Desmond [44] (Schrödinger Release 2025-1: Desmond Molecular Dynamics System, version 8.2, New York, NY, 2024. Maestro-Desmond Interoperability Tools, Schrödinger, New York, NY, 2025). The complex was prepared using the System Builder tool with the TIP3P water model and the OPLS 2005 force field. A 10 Å buffer was used to build an orthorhombic simulation box, and Na^+^ ions were used to neutralize the system. After energy minimization, the system underwent a multi-step relaxation protocol as follows:

- Step a: NVT (constant number of particles, volume, and temperature) simulation of Brownian dynamics at 300 K for 6250 ps, with restraints applied to non-hydrogen solute atoms.
- Step b: NVT simulation for 750 ps using a Langevin thermostat at 300 K, with rapid temperature relaxation and velocity resampling every 1 ps, maintaining restraints on non-hydrogen solute atoms.
- Step c: NPT (constant number of particles, pressure, and temperature) simulation for 750 ps at 300 K and 1 atm using the Langevin thermostat and barostat, applying slow pressure relaxation under the same restraints.
- Step d: NPT simulation for 750 ps with restrained non-hydrogen solute atoms, using dynamically assigned temperature and pressure (default: 300 K, 1 atm).
- Step e: Final NPT simulation for 1500 ps at 300 K and 1 atm with fast temperature and normal pressure relaxation constants.

Following equilibration, an MD run of 500 ns was performed under NPT conditions at 310 K and 1.01 bar, using a 2.0 fs integration timestep and a recording interval of 1.2 ps.

Analysis of the molecular dynamics trajectories was carried out with the Simulation Interaction Diagram tool included in the Schrödinger Suite.

### 3.5. Materials and Methods for Biological Evaluation

#### 3.5.1. Cell Line

The HeLa cell line was obtained from ATCC and cultured in DMEM (Euroclone) supplemented with 10% fetal bovine serum (Sigma-Aldrich), 2 mmol/L L-glutamine (Euroclone), and antibiotics: 100 μg/mL penicillin, 100 μg/mL streptomycin, and 250 ng/mL amphotericin B.

#### 3.5.2. MTT/CCK-8 Assay

Cell viability was evaluated using both the MTT and CCK-8 colorimetric assays. HeLa cells 2 × 10^4^ cells/well) were plated in 96-well microplates and treated with different concentrations of CAU for the indicated time periods, each condition performed in triplicate. After incubation, MTT (0.5 mg/mL; Merck) was added to each well, and cells were incubated for 4 h at 37 °C to allow formazan crystal formation. The culture medium was then carefully discarded, and the insoluble formazan was dissolved in isopropanol (Carlo Erba Reagents, Cornaredo, Italy). For the CCK-8 assay, the corresponding reagent was added to the wells, followed by an incubation phase that enabled the enzymatic conversion necessary for color development. Absorbance for each assay was then recorded at the appropriate wavelength using a TECAN Infinite M200 microplate reader (Tecan, Männedorf, Switzerland), and the resulting values were used to determine relative cell viability.

#### 3.5.3. PI Staining and Cell Cycle Analyses

The cell line HeLa was exposed to CAU 10 µM at varying time points. Following treatment, cells were harvested, centrifuged at 1200 rpm for 5 min, and then resuspended in 500 µL of propidium iodide buffer (1× PBS containing 2 mg/mL propidium iodide) for evaluating cell death. For the analysis of the cell cycle, cells were instead resuspended in 400 µL of a hypotonic solution composed of 1× PBS, 50 µg/mL PI, 0.1% sodium citrate, and 0.1% Triton X-100. Prior to data acquisition using a BD FACS-Celesta flow cytometer, samples were incubated at room temperature for 30 min. All assays were conducted in duplicate.

#### 3.5.4. Seahorse Assay

ATP production was investigated using a Seahorse XF24 Analyzer (Agilent Technologies, Santa Clara, CA, USA) with standard 24-well Seahorse plates. A Seahorse XF Real-Time ATP Rate Assay Kit (Agilent Technologies, #103592) was used to assess oxygen consumption rate (OCR), proton efflux rate (PER), and ATP production after HeLa cells were treated with CAU at 10 μM. Briefly, the sensor cartridge in the Seahorse XF Calibrant was hydrated in a non-CO_2_ incubator at 37 °C overnight. Subsequently, the cells were incubated at 37 °C for 1 h before analysis. Oligomycin (1.5 μM), rotenone (0.5 µM), and antimycin A (0.5 µM) were added sequentially at the indicated time points. Data was normalized by assessing cell viability with an MTT assay.

## 4. Conclusions

This study employed an MS-based functional proteomics approach to identify the molecular targets of the alkaloid caulerpin (CAU), a bioactive marine natural product recognized as a promising compound for drug discovery, thus providing insights into protein/ligand interactions.

The Drug Affinity Responsive Target Stability (DARTS) approach provided targets for the identification of three proteins potentially relevant in an anticancer context. In the present study, we focused on the detailed characterization of the complex formed between CAU and RUVBL1, a protein whose pivotal role in cancer biology is well established, while future research will explore interactions between CAU and the other two interactors.

Modulating RUVBL1 is a promising therapeutic approach, with ongoing efforts to find new interactors affecting its activity. It is a crucial metabolic enzyme involved in various biochemical processes strictly connected to tumour cell proliferation and survival, and it has already been studied as a potential therapeutic target. The following MS-based targeted limited proteolysis (t-LiP-MS) approach, along with in silico analysis, confirmed RUVBL1 as a specific partner of CAU and also provided relevant information on their mode of interaction, demonstrating that CAU binds RUVBL1 at the ATP-binding pocket, a conserved motif critical for ATP binding and hydrolysis.

This evidence suggested that CAU could affect RUVBL1’s enzymatic activity. On this basis, biological assays were performed to reveal CAU’s ability to modulate RUVBL1 activity and influence relevant cellular pathways. Metabolic analyses showed that CAU treatment significantly reduced cellular energy production and overall metabolic activity. This indicates a notable disruption of the main energy-producing pathways, pushing cells into low-energy or stressed conditions. This cytostatic profile suggests that CAU subtly suppresses cellular metabolism to limit tumor-cell proliferation and promote cell survival.

Given the current scientific interest in developing inhibitors or modulators of RUVBL1, the identification of this protein as a molecular target of CAU is a relevant achievement. It provides mechanistic insight into the action of CAU while simultaneously revealing a scaffold of outstanding significance in a well-established and actively explored therapeutic domain. The results provide a strong basis for considering CAU as a potential reference compound for targeting RUVBL1 in future drug discovery studies.

Therefore, this study demonstrates that functional proteomics complemented with biochemical and computational approaches is an effective analytical method to elucidate the mechanisms underlying natural product activity, underscoring the importance of continued exploration of the natural kingdom to identify valuable scaffolds with promising roles as lead compounds.

## Figures and Tables

**Table 1:** The table reports the abundance ratios calculated by Proteome Discoverer (PD) for RUVBL1 in the three DARTS experiments (ratios between RUVBL1 abundance in each sample treated with different CAU concentrations and untreated control). The increasing trend indicates that the amount of protein remaining after limited proteolysis rises with increasing CAU concentration, demonstrating a concentration-dependent protective effect of the molecule on the protein.

	Abundance Ratios		
	1 µM/ctrl	10 µM/ctrl	100 µM/ctrl
Replicate A	1.073	1.315	1.805
Replicate B	1.007	1.685	2.468
Replicate C	1.082	1.096	1.706

**Table 2:** t-LiP peptides with their Q1 and Q3 *m*/*z* values, their abbreviated sequence, and their fold change values, analyzed in triplicate, are reported. (*) All values were identified with a *p*-value lower than 0.05.

			Fold Change *	
Q1_mz	Q3_mz	Sequence	Treated	Untreated
775.39	200.10	A[379–400]K	2.48	10.12
540.82	684.36	A[65–76]K	2.56	17.43
765.94	940.58	Y[405–418]K	3.45	240.48
650.83	200.10	Q[34–46]R	2.90	4.42
615.84	260.19	E[47–57]K	2.28	5.05

## Data Availability

The original contributions presented in the study are included in the article; further inquiries can be directed to the corresponding author.

## Supplementary Materials

The following supporting information can be downloaded at: https://www.mdpi.com/article/10.3390/md24010037/s1, Table S1: List of other CAU’s protein targets identified across three DARTS experiments; Table S2: Parameters for the t-LiP-MS method, including the selected and monitored peptides of the RUVB-L1 protein, with their amino acid sequences, the corresponding *m*/*z* values for precursor (Q1) and fragment (Q3) ions, their respective charges, and MS parameters such as DP (declustering potential), EP (entrance potential), and CE (collision energy); Figure S1: CAU effect on HeLa cells’ viability. Cell viability was assessed by CCK-8 assay upon CAU treatment at the indicated doses and time points. Results were expressed as the percentage of cell viability relative to control (mean ± SD, *n* = 3); Figure S2: Effects of 10 µM CAU on the cell cycle. Representative image of the cell cycle of HeLa cells after 72 h of treatment with CAU. The percentage distribution of cells across the cell cycle phases was plotted in the graph below (mean ± SD, *n* = 2); Figure S3: Representative SDS–PAGE gel of one DARTS biological replicate. Each lane contains proteins treated with increasing amounts of CAU and subtilisin, except for the last lane, which contains proteins without CAU and without subtilisin (positive control). As suggested by the Coomassie-stained band intensities, proteins underwent different degrees of proteolytic digestion, consistent with the applied protease concentrations. Red lines indicate how the gel was subdivided and cut for subsequent in situ tryptic digestion.
