# Supplementary material for: Discovery of RUVBL1 as a Target of the Marine Alkaloid Caulerpin via MS-Based Functional Proteomics
Source: Mar Drugs. 2026 Jan 10;24(1):37. doi: 10.3390/md24010037 (PMC12842763; doi:10.3390/md24010037)
Supplement: Supplementary file 1 [file marinedrugs-24-00037-s001.zip › marinedrugs-3987638-supplementary.pdf]

Supporting Information

Discovery of RUVBL1 as a Target of the Marine Alkaloid Caulerpin via MS-Based Functional Proteomics

Alessandra Capuano [1], Gilda D'Urso [1], Lucia Capasso [2], Emilio Brancaccio [1], Erica Gazzillo [1], Marianna Carbone [3], Ernesto Mollo [3], Gianluigi Lauro [1], Maria Giovanna Chini [4], Giuseppe Bifulco [1], Angela Nebbioso [2] and Agostino Casapullo [1,*]

[1] Department of Pharmacy, University of Salerno, Via Giovanni Paolo II 132, 80084 Fisciano, Italy; acapuano@unisa.it (A.C.); gidurso@unisa.it (G.D.); embrancaccio@unisa.it (E.B.); egazzillo@unisa.it (E.G.); glauro@unisa.it (G.L.); bifulco@unisa.it (G.B.)
[2] Department of Precision Medicine, University of Campania "Luigi Vanvitelli", Via De Crecchio 7, 80138 Naples, Italy; lucia.capasso2@unicampania.it (L.C.); angela.nebbioso@unicampania.it (A.N.)
[3] Institute of Biomolecular Chemistry, National Research Council of Italy, Via Campi Flegrei 34, 80078 Pozzuoli, Italy; marianna.carbone@cnr.it (M.C.); emollo@icb.cnr.it (E.M.)
[4] Department of Biosciences and Territory, University of Molise, Contrada Fonte Lappone, 86090 Isernia, Italy; mariagiovanna.chini@unimol.it
* Correspondence: casapullo@unisa.it

Table of Contents

Table S1. List of other CAU's protein targets identified across three DARTS experiments. 3

Table S2 Parameters for the t-LiP-MS method, including the selected and monitored peptides of the RUVB-L1 protein, with their amino acid sequences, the corresponding *m/z* values for precursor (Q1) and fragment (Q3) ions, their respective charges, and MS parameters such as DP (declustering potential), EP (entrance potential), and CE (collision energy). 4

Figure S1. CAU effect on HeLa cells' viability. Cell viability was assessed by CCK-8 assay upon CAU treatment at the indicated doses and time points. Results were expressed as the percentage of cell viability relative to control (mean ± SD, n = 3). 4

Figure S2. Effects of 10 μM CAU on the cell cycle. Representative image of the cell cycle of HeLa cells after 72 hours of treatment with CAU. The percentage distribution of cells across the cell cycle phases was plotted in the graph below (mean ± SD, n = 3). 5

.... 6

Figure S3. Representative SDS–PAGE gel of one DARTS biological replicate. Each lane contains proteins treated with increasing amounts of CAU and subtilisin, except for the last lane, which contains proteins without CAU and without subtilisin (positive control). As suggested by the Coomassie-stained band intensities, proteins underwent different degrees of proteolytic digestion, consistent with the applied protease concentrations. Red lines indicate how the gel was subdivided and cut for subsequent in situ tryptic digestion 6

Table S1. List of other CAU's protein targets identified across three DARTS experiments.

	Replicate A				*Replicate B*				*Replicate C*			
**Description**	1uM/ctrl	10uM/ctrl	100uM/ctrl	lys/ctrl	1uM/ctrl	10uM/ctrl	100uM/ctrl	lys/ctrl	1uM/ctrl	10uM/ctrl	100uM/ctrl	lys/ctrl
**Kinetochore protein Spc24 (Fragment) (K7ESQ2)**	n.d.	10,2	30,8	100	n.d.	21,2	17,7	100	31,6	54,5	87,5	100
**Serine/threonine-protein phosphatase 2A 55 kDa regulatory subunit B alpha isoform (P63151)**	8,3	13,4	20,5	100	0,8	6,0	54,7	100	44,8	49,3	55,7	100

Table S2 Parameters for the t-LiP-MS method, including the selected and monitored peptides of the RUVB-L1 protein, with their amino acid sequences, the corresponding *m/z* values for precursor (Q1) and fragment (Q3) ions, their respective charges, and MS parameters such as DP (declustering potential), EP (entrance potential), and CE (collision energy).

Q1_mz	Charge Q1	Q3_mz	Charge Q3	Sequence	DP	EP	CE
650.33	3	895.96	2	GTEDITSPHGIPLDLLDR	80	15	33.6
775.39	3	200.10	1	AQTEGINISEEALNHLGEIGTK	80	15	40.0
570.27	3	267.10	1	EHVEEISELFYDAK	80	15	29.5
693.40	2	286.17	1	TALALAIAQELGSK	80	15	36.0
844.47	2	1016.58	1	ALESSIAPIVIFASNR	80	15	42.6
540.82	2	684.36	1	AVLLAGPPGTGK	80	15	29.3
569.78	2	231.09	1	TEVLMENFR	80	15	30.5
765.94	2	940.58	1	YSVQLLTPANLLAK	80	15	39.2
650.83	2	200.10	1	QAASGLVGQENAR	80	15	34.1
530.28	2	218.15	1	GLGLDESGLAK	80	15	28.8
638.84	2	326.17	1	LDPSIFESLQK	80	15	33.6
615.84	2	260.19	1	EACGVIVELIK	80	15	32.6

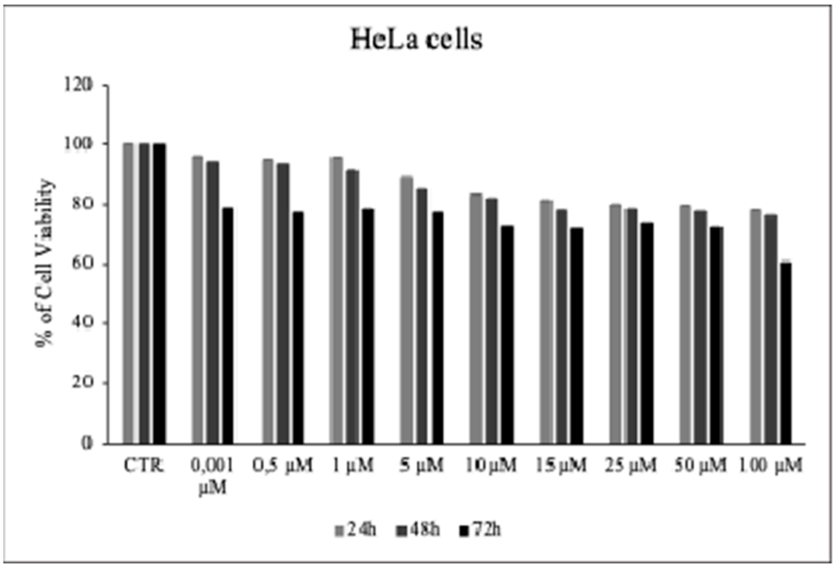

Figure S1. CAU effect on HeLa cells' viability. Cell viability was assessed by CCK-8 assay upon CAU treatment at the indicated doses and time points. Results were expressed as the percentage of cell viability relative to control (mean ± SD, n = 3).

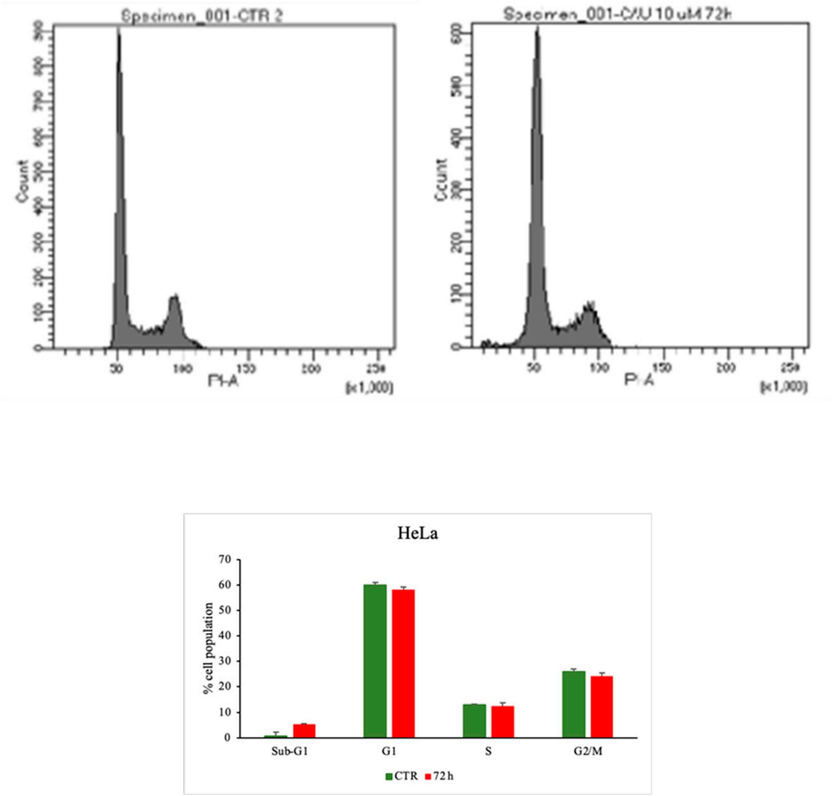

Figure S2. Effects of 10 μM CAU on the cell cycle. Representative image of the cell cycle of HeLa cells after 72 hours of treatment with CAU. The percentage distribution of cells across the cell cycle phases was plotted in the graph below (mean ± SD, n = 3).

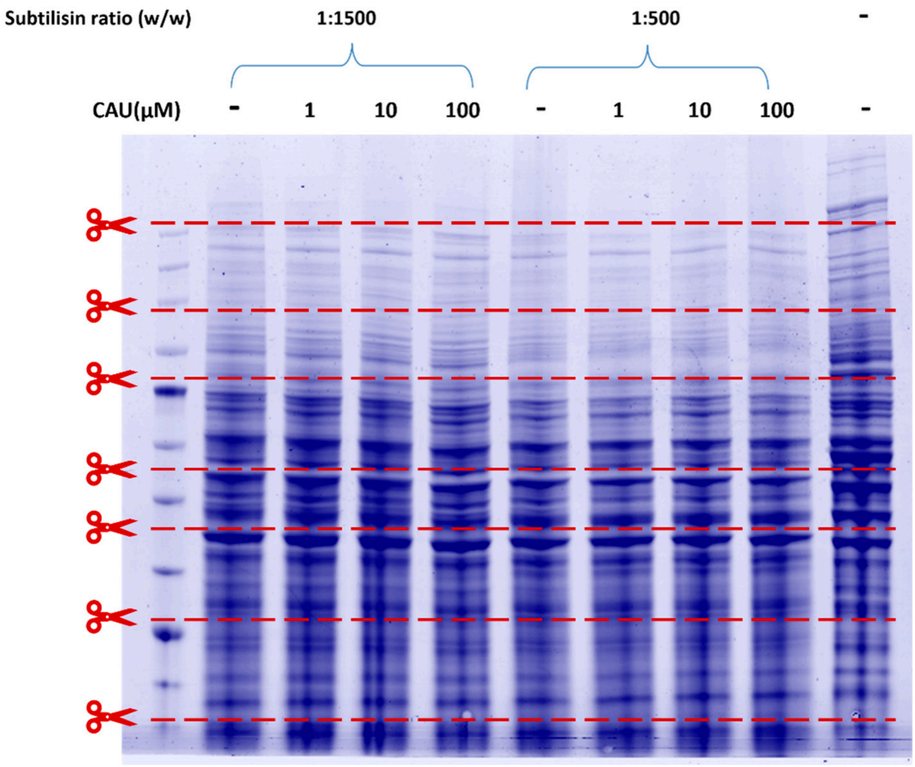

Figure S3. Representative SDS–PAGE gel of one DARTS biological replicate. Each lane contains proteins treated with increasing amounts of CAU and subtilisin, except for the last lane, which contains proteins without CAU and without subtilisin (positive control). As suggested by the Coomassie-stained band intensities, proteins underwent different degrees of proteolytic digestion, consistent with the applied protease concentrations. Red lines indicate how the gel was subdivided and cut for subsequent in situ tryptic digestion.
